# Development of a Cytogenetic Double-Hit Model for Survival Prediction in Multiple Myeloma

**DOI:** 10.3390/cancers17162703

**Published:** 2025-08-20

**Authors:** Chenxing Du, Jian Cui, Jingyu Xu, Wenqiang Yan, Lingna Li, Weiwei Sui, Shuhui Deng, Shuhua Yi, Yan Xu, Chengwen Li, Jiawei Zhao, Dehui Zou, Lugui Qiu, Gang An

**Affiliations:** 1 State Key Laboratory of Experimental Hematology, National Clinical Research Center for Blood Diseases, Haihe Laboratory of Cell Ecosystem, Institute of Hematology & Blood Diseases Hospital, Chinese Academy of Medical Sciences & Peking Union Medical College, Tianjin 300020, China; duchenxing@ihcams.ac.cn (C.D.); cuijian@ihcams.ac.cn (J.C.); xujingyu@ihcams.ac.cn (J.X.); yanwenqiang@ihcams.ac.cn (W.Y.); lilingna@ihcams.ac.cn (L.L.); suiweiwei@ihcams.ac.cn (W.S.); dengshuhui@ihcams.ac.cn (S.D.); yishuhua@ihcams.ac.cn (S.Y.); xuyan1@ihcams.ac.cn (Y.X.); lichengwen@ihcams.ac.cn (C.L.); zhaojiawei@ihcams.ac.cn (J.Z.); zoudehui@ihcams.ac.cn (D.Z.); qiulg@ihcams.ac.cn (L.Q.); 2 Tianjin Institutes of Health Science, Tianjin 301600, China; 3 LeBow Institute for Myeloma Therapeutics and Jerome Lipper Multiple Myeloma Center, Dana-Farber Cancer Institute, Harvard Medical School, Boston, MA 02215, USA

**Keywords:** multiple myeloma, double hit, interphase fluorescence in situ hybridization, cytogenetic abnormalities, prognosis

## Abstract

**Background:** High-risk chromosomal abnormalities (HRCAs) detected by fluorescence in situ hybridization (FISH) have a well-established adverse prognostic impact in multiple myeloma (MM). It is increasingly recognized that the coexistence of two or more HRCAs identifies a particularly poor-risk subgroup, often referred to as double- or multiple-hit MM. However, there is currently no consensus on its definition. **Methods:** We retrospectively analyzed a multicenter cohort of 1122 newly diagnosed MM patients from 2008 to 2019. Double-hit MM was defined as the coexistence of at least two of the following four HRCAs: t(14;16), gain(1q), del(17p), and del(1p). Based on this definition, we constructed a novel prognostic model, the HBDH (Institute of Hematology & Blood Diseases Hospital) double-hit model, and assessed its prognostic value for progression-free survival (PFS) and overall survival (OS). **Results:** According to the HBDH model, double-hit patients showed significantly inferior outcomes compared to non-double-hit patients, with median PFS of 20.6 vs. 53.3 months (*p* < 0.001) and median OS of 40.2 vs. 84.2 months (*p* < 0.001). The addition of del(13q), t(4;14), or t(11;14) did not improve the prognostic performance of the model. Importantly, the HBDH model was independent of the International Staging System (ISS), elevated LDH, and advanced age. **Conclusions**: The HBDH double-hit model identifies a subset of ultra-high-risk MM patients carrying at least two major HRCAs, providing a simple and robust framework for prognostic stratification and a potential reference for future biologically driven treatment approaches.

## Simple Summary

Accurate risk stratification in multiple myeloma (MM) is critical for optimizing therapy. In our study, we analyzed a large cohort of newly diagnosed MM patients with fluorescence in situ hybridization (FISH) data to investigate the prognostic implications of various high-risk cytogenetic abnormalities (HRCAs). We systematically compared multiple definitions of double-hit MM and identified a simplified model comprising four key HRCAs with the strongest and most independent prognostic value. Our findings demonstrate that the co-occurrence of two or more of these specific HRCAs defines a distinct double-hit MM subgroup with significantly poorer survival, even in the era of novel therapies. This model not only refines risk prediction but also reflects the cumulative biological impact of complex cytogenetic lesions. By establishing a more practical and biologically meaningful framework for defining high-risk MM, our study provides valuable guidance for both clinical management and future research into targeted therapeutic strategies for this aggressive disease subset.

## 1. Introduction

More precise estimation of prognosis for patients with multiple myeloma (MM) can help with upfront identification of those who may relapse early, enabling the possibility of intervening pre-emptively to maintain long-term remission. Furthermore, identifying disease subgroups with targetable tumor dependencies can also guide biologically driven treatment [1]. Since drivers of cytogenetic abnormalities (CAs) underlie the key hallmarks of high-risk disease states, high-risk CAs (HRCAs) are considered the most important prognostic factor for MM [2,3]. A number of CAs, notably t(4;14), t(14;16), deletion 17p (del(17p)), and gain of 1q (gain(1q)), have been integrated into the Revised International Staging System (R-ISS) [4] and the Second Revision of the International Staging System (R2-ISS) [5].

Among patients with HRCAs, a distinct population classified as “double-hit” MM demonstrates an exceptionally high risk of disease progression and significantly worse survival outcomes [6,7,8]. This group of double-hit MM patients includes (1) patients with biallelic alterations in critical genes, such as biallelic inactivation of the *TP53* (del(17p) with *TP53* mutation most of the time) or biallelic deletion of 1p32 (del(1p32)) [9,10], and (2) patients carrying any two or more HRCAs, with those harboring three or more such HRCAs also referred to as “triple-hit” myeloma [8,11]. More recently, the International Myeloma Society (IMS) has presented a new Consensus Genomic Staging (CGS) system for high-risk MM patients [12]. In this new consensus definition, MM patients with biallelic del(1p), or any two of the intermediate-risk CAs, are defined as high-risk, which also highlights the cumulative effect of multiple CAs. Furthermore, the definition of double-hit MM may also help explain part of the heterogeneity in clinical characteristics, treatment response, and prognosis observed in historical HRCA patients [13,14,15]. However, several questions are still debated. The first is which HRCA should be included in the double-hit model, and whether different combinations of HRCAs carry distinct prognostic implications [16]. In a meta-analysis of 1905 trial patients with molecular profiles, MM patients with co-occurrence of at least two HRCAs, including t(4;14), t(14;16), t(14;20), del(17p), and gain(1q), were defined as double-hit MM [7]. The second question, related to the first one, concerns whether gain(1q) or del(1p) should be included in the double-hit model, as the adverse prognostic significance of these two CAs has been confirmed in multiple studies [10,17,18].

Co-occurrence of multiple CAs is quite common in MM, and the cytogenetic lesions may confer cumulative adverse prognostic significance. In this study, we analyzed a large cohort of newly diagnosed MM (NDMM) with fluorescence in situ hybridization (FISH) examination of HRCAs. We intended to screen out the most decisive and irreplaceable HRCAs and develop a simplified HRCA model for defining double-hit MM. The analysis identified a four-HRCA double-hit model after comparing various double-hit definitions in the era of novel therapies.

## 2. Materials and Methods

### 2.1. Data Source and Study Population

This study was carried out using the MM database of the National Longitudinal Cohort of Hematological Diseases (NICHE, NCT04645199), collected from January 2008 to December 2019 at the Institute of Hematology & Blood Diseases Hospital, Chinese Academy of Medical Science & Peking Union Medical College (IH & BDH, CAMS & PUMC). As shown in Supplemental Figure S1, a total of 1122 NDMM patients with at least one CA datapoint were identified and enrolled in this study. Among them, 984 patients had the required HRCAs data, including t(4;14), t(14;16), del(17p), and gain(1q). A total of 757 patients with complete FISH test data, which, in addition to the aforementioned abnormalities, also included t(14;20) and del(1p), were included in the double-hit model (Supplemental Figure S1). All patients were assigned to either immunomodulating drug-based or proteasome inhibitor-based induction, as previously described [19]. Specifically, patients were assigned to either thalidomide-based regimens (TAD: thalidomide, adriamycin, dexamethasone, or TCD: thalidomide, cyclophosphamide, dexamethasone) or bortezomib-based regimens (BCD: bortezomib, cyclophosphamide, dexamethasone, or PAD: bortezomib, adriamycin, dexamethasone). In addition, some patients received a proteasome inhibitor plus immunomodulating drug-based induction, most commonly the VRD regimen (bortezomib, lenalidomide, and dexamethasone). After four to six cycles of induction and achieving a minimum partial response, patients underwent either upfront autologous stem cell transplant (ASCT) if eligible, or two additional cycles of consolidation treatment if transplant-ineligible or by patient choice. All the patients provided informed consent in compliance with the Declaration of Helsinki. The study received approval from the local institutional ethics committees of the IH & BDH, CAMS & PUMC (Certificate: IIT2020023-EC-1).

### 2.2. FISH Testing

Bone marrow (BM) samples were collected from NDMM patients, and CD138+ plasma cells were then enriched using CD138 magnetic beads (Miltenyi Biotec, Paris, France), enabling a post-sorting purity higher than 90%. The samples were then analyzed on the purified PCs, and a total of 200 interphase nuclei were tested. FISH panel included the following probes: RB1/LAMP1, TP53, IgH dual color break-apart rearrangement, dual-color, dual-fusion probes for t(4;14)(p16;q32), t(11;14)(q13;q32), t(14;16)(q32;q23) and t(14;20)(q32;q12) (Abbott Molecular Des Plaines, IL, USA), and CKS1B/CDKN2C (Oxford Gene Technology, Kidlington, UK). Unless specifically stated, the cut-off value was 20% for chromosome deletion or gain, and 10% for chromosome translocation.

High-risk cytogenetic abnormalities (CAs) were those defined by the R-ISS [4]: del(17p), t(4;14), and t(14;16); by the Mayo Stratification for Myeloma and Risk-Adapted Therapy 3.0 (mSMART 3.0) [8]: t(4;14), t(14;16), t(14;20), del(17p), *TP53* mutation, and gain(1q); and by the NCRI model [7]: t(4;14), t(14;16), t(14;20), del(17p), gain(1q), and del(1p32). Patients with at least two HRCAs were defined as double-hit MM.

### 2.3. Statistical Analysis

Overall survival (OS) was calculated from the date of initial therapy to the date of death or the last follow-up. Progression-free survival (PFS) was calculated from the date of initial therapy to the date of death, first documented disease progression, or the last follow-up, whichever happened first. Survival curves were plotted using the Kaplan–Meier method and compared by the two-sided log-rank test. A multivariate Cox proportional-hazards model was developed to assess the variables with significant effects on PFS and OS based on univariate analysis. Explained variation for survival models was calculated using the methods described by Gleiss et al. [20], which quantify the proportion of variation in survival time accounted for by HRCAs. The performance of the HBDH model was evaluated through Kaplan–Meier survival curves, hazard ratio (HR) estimation, and Harrell’s concordance index (C-statistic), with the latter assessed using bootstrap resampling with 1000 iterations. The statistical significance of categorical variables was calculated using Chi-Square or Fisher’s exact tests.

The statistical significance of continuous variables was calculated using the Kruskal–Wallis test. All statistical analyses were conducted using SPSS (version 26.0; IBM, Chicago, IL, USA) and R (version 4.3.0; R Foundation, Vienna, Austria).

## 3. Results

In this study, a total of 1122 NDMM patients diagnosed at the IH & BDH, CAMS & PUMC, and identified to have at least one CA were included (Supplemental Figure S1). The median follow-up time for the entire cohort was 50.0 (range: 2–138) months. The median PFS and OS for the 1122 patients were 37.7 (34.1–41.3) months and 65.8 (60.2–71.4) months, respectively. Moreover, 685 (61.1%) patients received proteasome inhibitor-based therapy, 149 (13.3%) patients received proteasome inhibitor plus immunomodulating drug-based induction treatment, and 263 (23.4%) patients received immunomodulating drug-based induction treatment. In addition, 22.2% (249/1122) of patients received first-line ASCT (Supplementary Table S1). 

### 3.1. Prognostic Value of CAs in NDMM

We first assessed the prognostic impact of various CAs on survival outcomes in NDMM patients. In the univariate analysis, patients with t(14;16), del(13q), del(17p), gain(1q), or del(1p) exhibited significantly worse PFS and OS compared to those without these abnormalities. Specifically, the median PFS values for patients with the aforementioned CAs were 18.1 vs. 39.8, 32.0 vs. 46.0, 21.2 vs. 46.0, 30.0 vs. 48.1, and 29.0 vs. 40.2 months, respectively. Similarly, the median OS values for these patients were 40.0 vs. 68.9, 52.8 vs. 73.6, 44.2 vs. 67.3, 50.0 vs. 83.2, and 39.5 vs. 71.4 months, respectively (Table 1). In the multivariate analysis, only del(17p) and gain(1q) were independently associated with adverse effects on both PFS and OS (Table 2; Supplemental Table S2).

**Figure 1:**
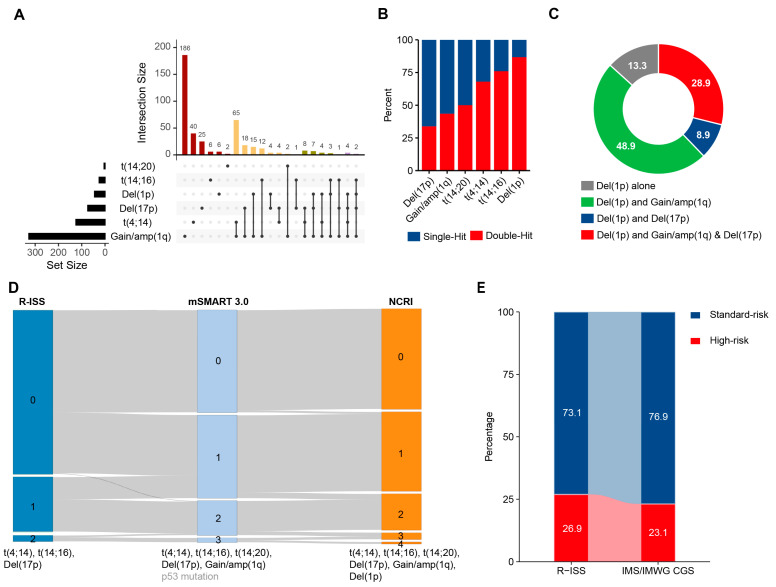
Co-occurrence patterns of CAs in patients with NDMM. (**A**). Upset plot of CAs detected by FISH in 957 NDMM patients, color-coded by cytogenetic co-occurrence patterns in MM. (**B**). Bar plot showing the proportion of patients with distinct multi-hit statuses among those carrying each HRCA in NDMM. (**C**). Pie chart displaying the co-occurrence of other genetic abnormalities among patients with del(1p32) (**D**). Comparison of the distribution of NDMM patients across different double-hit models. (**E**). Sankey diagram showing the distribution and migration of patients’ risk status between two different risk models, R-ISS and IMWG/IMS CGS consensus definition.

The most common combinations of high-risk CAs included gain(1q) with one of the following: t(4;14) (54.0%, 81/150), del(17p) (26.7%, 40/150), del(1p) (23.3%, 35/150), and t(14;16) (12.0%, 18/150) (Figure 1A). Notably, among patients with del(1p32), 86.7% harbored one or more additional high-risk cytogenetic abnormalities in addition to del(1p32) (Figure 1B). The most common combinations included del(1p32) with gain(1q) (48.9%), del(1p32) with del(17p) (8.9%), and del(1p32) with both gain(1q) and del(17p) (28.9%) (Figure 1C).

### 3.2. Comparison Between Different Double-Hit Models

We then studied the differences between currently available double-hit models for MM. Per R-ISS [4], Mayo Stratification for Myeloma and Risk-Adapted Therapy 3.0 (mSMART 3.0) [8], or NCRI models [7], the percentage of patients with no HRCA varied from 73.1% (553/757), 45.7% (346/757), to 44.8% (339/757). Furthermore, 24.3% (184/757), 37.0% (280/757), and 35.4% (268/757) of patients were classified as single-hit MM, and 2.7% (20/757), 15.5% (117/757), and 19.8% (150/757) of patients were classified as double-hit MM by the three models. Compared to R-ISS, mSMART 3.0 redirected 37.1% (205/553) of patients with no HRCA into the single-hit group and redirected 59.2% (109/184) of single-hit patients into the double-hit group. Additionally, compared to mSMART 3.0, NCRI redirected 3.4% (26/757) of patients into a higher risk degree (Figure 1D). Furthermore, when comparing the definitions of HRCAs in R-ISS and the newly updated IMWG/IMS CGS HRCA consensus definition, slightly fewer patients were classified as high-risk MM under the IMWG/IMS CGS criteria, with proportions of 26.9% and 23.1%, respectively (Figure 1E; Supplemental Figure S2).

**Figure 2:**
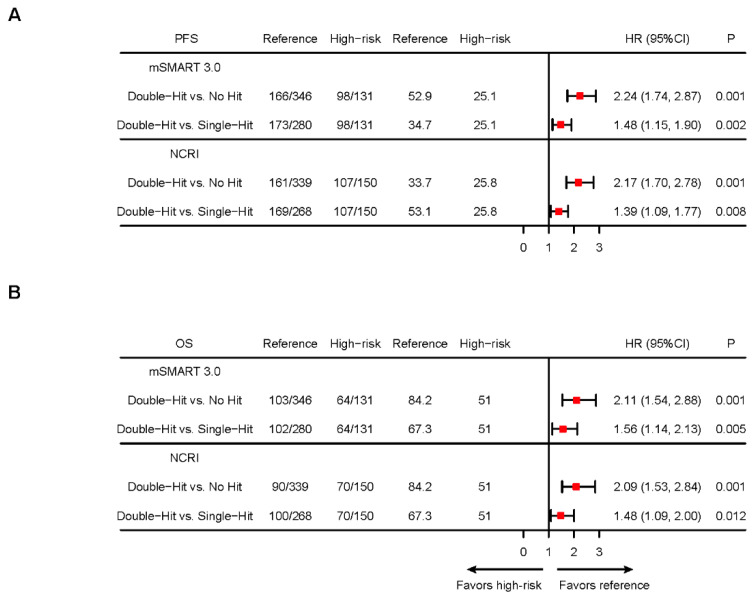
Kaplan–Meier analysis of multi-hit MM defined by different standards. (**A**). Forest plots of hazard ratios (HRs) for median PFS in patients with different multi-hit statuses defined by mSMART 3.0 or NCRI models (**B**). Forest plots of HRs for median OS in patients with different multi-hit statuses defined by mSMART 3.0 or NCRI models. NCRI HRCAs include t(4;14), t(14;16), t(14;20), del(17p), gain(1q), and del(1p32). mSMART 3.0 HRCAs include t(4;14), t(14;16), t(14;20), del(17p), and gain(1q). The mSMART 3.0 model excluded *TP53* mutation data due to partial unavailability in this cohort.

To further clarify the prognostic significance of different double-hit models, we compared the PFS and OS of patients with different HRCA numbers separately. Our results demonstrated that the number of HRCAs holds significant prognostic value for MM patients, regardless of the model used. According to the mSMART 3.0 model, PFS and OS for patients with no HRCAs, single-hit, or double-hit MM were 52.9, 34.7, and 25.1 months (*p* < 0.001) and 84.2, 67.3, and 51.0 months (*p* < 0.001), respectively. According to the NCRI model, the PFS and OS for patients with no HRCAs, single-hit, or double-hit MM were 53.1, 33.7, and 25.8 months (*p* < 0.001) and 84.2, 67.3, and 51.0 months (*p* < 0.001), respectively (Figure 2A,B; Supplemental Figure S3).

The primary distinction between the mSMART 3.0 and NCRI models lies in the inclusion of del(1p32) in the NCRI model. To explore this further, we analyzed the impact of del(1p32) within each HRCA subgroup defined by the mSMART 3.0 model. Our findings revealed that del(1p32) seldom exerted an additional adverse effect on survival across HRCA subgroups, except in patients who lacked any other HRCA (Supplemental Figure S4).

### 3.3. Development of the HBDH Double-Hit Model

We then sought to develop a double-hit model for MM that incorporates the fewest HRCA while achieving optimal prognostic predictive performance. We first examined del(13q), t(11;14), and t(4;14), three CAs that, based on our previous analyses, were not associated with adverse outcomes in patients with MM. In our cohort, 43.2% (327/757) of patients had del(13q). Among them, 23.9% (78/327) had no other HRCAs, 38.2% (125/327) had one HRCA, and 37.9% (124/327) had at least two HRCAs. Del(13q) was significantly associated with t(4;14), t(14;16), del(17p), gain(1q), and del(1p), but was mutually exclusive with t(11;14) (Supplemental Table S3). Stratification by the number of HRCAs revealed no significant differences in PFS or OS based on del(13q) status (Supplemental Figure S5).

For t(11;14), mutual exclusivity with other IgH translocations indicates its presence excludes high-risk IgH translocations such as t(4;14), t(14;16), or t(14;20). Compared with patients without other high-risk IgH translocations, patients with t(11;14) had similar PFS or OS (median PFS: 40.9 vs. 41.0 months; median OS: 67.3 vs. 73.0 months; Supplemental Figure S6A,B). Patients with t(11;14) were less likely to harbor secondary CAs, such as del(17p) (5.0% vs. 10.8%, *p* = 0.047) or gain(1q) (34.7% vs. 46.1%, *p* = 0.022; Supplemental Table S4). Stratification by the presence of secondary HRCAs showed that t(11;14) neither increased risk nor provided a protective benefit (Supplemental Figure S6C,D).

The association of t(4;14) with poor prognosis was not observed in NDMM patients in our cohort (Supplemental Figure S7A,B), except in those receiving immunomodulating drug-based induction therapy (Supplemental Figure S7C,D). Additionally, t(4;14) did not confer additional risk when stratified by other HRCAs such as t(14;16), gain(1q), del(1p), or del(17p) (Supplemental Figure S7E,F).

**Figure 3:**
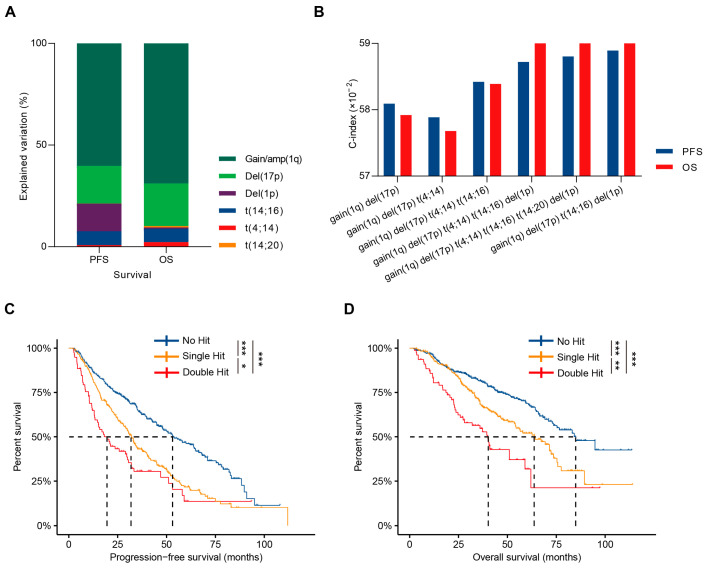
Development of the HBDH double-hit model. (**A**). Contribution of specific HRCAs to the explained variation in PFS and OS in NDMM patients. (**B**). The C-index evaluation of HRCA combinations. (**C**,**D**). Kaplan–Meier curves in NDMM patients with no HRCA, single-hit, or double-hit MM defined by the HBDH double-hit model. * *p* < 0.05, ** *p* < 0.01, *** *p* < 0.001, using two-sided log-rank test.

When analyzing the explained variation of all HRCAs, the top four contributors to survival were gain(1q), del(17p), del(1p), and t(14;16), forming the HBDH model developed by the Institute of Hematology & Blood Diseases Hospital (Figure 3A). The HBDH model demonstrated a concordance index of 0.59 for both PFS and OS, comparable to the NCRI model and a model incorporating all six HRCAs (Figure 3B).

The HBDH model stratified MM patients into three groups: no hit, single-hit, and double-hit, based on the number of HRCAs, with no hit indicating the absence of HRCAs, single-hit indicating one HRCA, and double-hit indicating two HRCAs, showing significant prognostic differences. Median PFS values were 53.3, 32.0, and 20.6 months (*p* < 0.001, Figure 3C), and median OS values were 84.2, 64.2, and 40.2 months (*p* < 0.001, Figure 3D) for no hit, single-hit, and double-hit MM patients, respectively. Patient-related risk factors, such as aging and poor performance status, were similar across subgroups; however, the number of HBDH HRCAs correlated positively with LDH elevation (*p* = 0.002), ISS stage 3 (*p* < 0.001), and other high-risk karyotype features. While the use of proteasome inhibitor-based and first-line ASCT was consistent across groups, patients with more HBDH-defined HRCAs faced a higher risk of early progression or death (*p* < 0.001, Table 3). Multivariate analysis, accounting for aging, LDH abnormalities, ISS stage, and the number of HRCAs, confirmed that the HBDH double-hit model can independently predict both PFS and OS (Table 4).

## 4. Discussion

Prior sequencing studies of MM revealed that certain CAs more often tend to co-occur; for example, the combination of t(4;14) and del(13q), and *TP53* mutation and del(17p) [21,22,23]. More recent studies have increasingly demonstrated that the cumulative prognostic impact is induced by associations multiple HRCAs [24,25,26]. Double-hit MM, defined as the presence of at least two HRCAs within a patient, is now considered a subset of ultra-high-risk myeloma [2]. However, there is currently no consensus on the definition of double-hit MM. In this study, we constructed the HBDH double-hit model based on four HRCAs, including gain(1q), del(17p), del(1p), and t(14;16), by comparing the explained variation of all included HRCAs and the C-index of various double-hit model combinations.

Inconsistent definitions of double-hit MM pose a significant challenge to systematic comparisons between clinical trials. A meta-analysis of the MRC Myeloma IX and NCRI Myeloma XI trials revealed that patients with double-hit MM, defined as the co-occurrence of at least two adverse lesions, including t(4;14), t(14;16), t(14;20), del(17p), del(1p), and gain(1q), experienced especially poor prognosis, with HRs for PFS and OS of 2.23 and 2.67, respectively [7]. This analysis represents one of the largest studies focusing on the additional risk of multiple HRCAs in NDMM patients, demonstrating that double-hit MM is associated with significantly worse outcomes. Interestingly, our study found no significant prognostic impact of t(4;14), diverging from the Myeloma IX and XI data. One possible explanation is that most of our patients received first-line bortezomib-based induction therapy, which may have mitigated the adverse effects of t(4;14). Meanwhile, the mSMART 3.0 defines double-hit MM as the presence of any two HRCAs and triple-hit MM as the presence of at least three, including t(4;14), t(14;16), t(14;20), del(17p), *TP53* mutation, and gain(1q) [8]. Survival analysis indicated that double-hit patients had median PFS and OS of 14.0 and 28.6 months, respectively. Given that many studies have found del(1p) to be associated with inferior outcomes in MM, it would be reasonable to include del(1p) in the mSMART 3.0 double-hit model. More recently, the IMS/IMWG CGS system has been proposed to provide a unified definition of high-risk MM, incorporating both classical cytogenetic abnormalities and next-generation sequencing-based markers [12]. This framework highlights the prognostic importance of bi-allelic *TP53* inactivation and gain(1q) and underscores the need to standardize risk definitions across studies.

HRCA detection techniques can vary widely in sensitivity and specificity [27,28]. The Myeloma Genome Project (MGP), using next-generation sequencing, defines double-hit MM as either bi-allelic *TP53* inactivation or amplification of 1q21 combined with International Staging System (ISS) stage 3 [6]. Double-hit MM comprised 6.1% of their cohort and had the poorest prognosis, with median PFS and OS of 15.4 and 20.7 months, respectively. Although there is ongoing debate about whether bi-allelic *TP53* inactivation carries additional risk beyond del(17p) alone [9,29], our study confirmed that del(17p) combined with another HRCA was associated with adverse outcomes in MM. Another study using targeted sequencing compared different MM double-hit models, including the NCRI double-hit model, bi-allelic *TP53* inactivation, and the mSMART 3.0 model, showed that double-hit MM accounted for 10.9%, 6.5%, and 15.2% of NDMM patients, respectively [30]. Whole-genome sequencing reported that 30.8% of NDMM and 44.3% of relapsed/refractory MM patients had double-hit events, with gain(1q) and loss of heterozygosity at 17p being the most frequent double-hit abnormalities in the relapsed/refractory cohort [31]. Our findings suggest that the combination of del(17p) and gain(1q) was among the most critical prognostic factors in MM.

By comparing the prognostic predictive efficacy of the HBDH double-hit model with that of other double-hit models, we found that the HBDH double-hit model demonstrated comparable ability to identify high-risk patients. Among the factors analyzed, gain(1q), del(17p), del(1p), and t(14;16) accounted for the largest variation in PFS and OS differences. By stratifying patients into no hits, single hits, or double hits, the HBDH double-hit model can clearly distinguish outcomes with distinct multi-hit statuses. Notably, the median PFS and OS of double-hit MM, as defined by the HBDH model, coincidentally aligned with the concept of early relapse or early death in MM. Additionally, this study demonstrated that t(4;14), t(11;14), and del(13q) did not contribute additional risk to either single-hit or double-hit patients. Importantly, the HBDH model was shown to be independent of ISS stage, LDH levels, and age, suggesting its potential utility as part of a comprehensive prognostic prediction tool for MM patients.

## 5. Conclusions

In conclusion, our study confirmed that the number of HRCAs is negatively correlated with survival, with double hits posing additional risks for MM patients. We developed a model to assess the risk in single-hit and double-hit patients, finding that a four-HRCA model could simply stratify patients into different risk subgroups. Notably, the adverse prognostic impact of t(4;14) appears to diminish in the era of bortezomib-based therapy. Similarly, t(11;14) and del(13q) may not contribute additional risk to double-hit MM patients. Our findings suggest that double-hit MM is associated with a high risk of early progression or death. Further validation of this model in prospective studies is needed to confirm its prognostic utility and refine risk stratification for MM patients.

## Figures and Tables

**Table 1:** PFS and OS of 1122 newly diagnosed MM patients with each cytogenetic aberration.

Cytogenetic Aberrations	Incidence Rate	PFS			OS		
		Negative	Positive	*p*	Negative	Positive	*p*
t(11;14)	169/994 (17.0%)	37.8	35.6	0.550	68.9	63.9	0.298
t(4;14)	175/1013 (17.3%)	39.4	34.2	0.021	71.0	59.4	0.122
t(14;16)	34/1011 (3.4%)	39.8	18.1	0.001	68.9	40.0	0.005
t(14;20)	4/992 (0.4%)	37.7	20.0	0.691	68.7	56.4	0.922
t(14; undefined) ^1^	122/974 (12.5%)	37.8	34.9	0.893	66.4	77.5	0.076
del(13q)	481/1105 (43.5%)	46.0	32.0	<0.001	73.6	52.8	<0.001
del(17p)	94/1109 (8.5%)	39.8	21.2	<0.001	67.3	44.2	0.006
gain(1q)	503/1096 (45.9%)	48.1	30.0	<0.001	83.2	50.0	<0.001
del(1p)	53/886 (6.0%)	40.2	29.0	0.001	71.4	39.5	<0.001
Hypodiploidy ^2^	67/883 (7.6%)	39.0	18.9	<0.001	66.4	27.1	<0.001
CK ^3^	91/883 (11.9%)	39.1	22.0	<0.001	66.4	34.4	<0.001

^1.^ Patients with IgH translocation and negative results for t(11;14), t(4;14), t(14;16) or t(14;20). ^2.^ Hyperdiploidy was defined as >46 chromosomes by metaphase cytogenetics, typically showing gains of multiple odd-numbered chromosomes. ^3.^ CK, complex karyotype, defined as the presence of two or more cytogenetic abnormalities identified through conventional metaphase cytogenetics.

**Table 2:** Multivariate survival analysis of high-risk cytogenetic aberrations in 757 patients with complete CA data, including t(4;14), t(14;16), t(14;20), del(17p), gain(1q), and del(1p).

Cytogenetic Aberrations	PFS				OS	
	HR	95% CI	*p*	HR	95% CI	*p*
t(4;14)	1.12	0.88–1.43	0.356	1.07	0.78–1.47	0.666
t(14;16)	1.45	0.92–2.29	0.107	1.44	0.83–2.48	0.194
t(14;20)	1.35	0.43–4.22	0.607	1.09	0.27–4.40	0.902
del(17p)	1.55	1.15–2.09	0.004	1.49	1.03–2.14	0.033
gain(1q)	1.66	1.36–2.03	<0.001	1.62	1.25–2.08	<0.001
del(1p)	1.10	0.75–1.61	0.622	1.49	0.97–2.30	0.069

**Table 3:** Clinical features of patients with zero, one, or at least two HRCAs. HRCAs included t(14;16), del(17p), del(1p), and gain(1q) (HBDH double-hit model).

	No Hit	One Hit	Double Hit	*p*
Patient number	372 (49.1%)	304 (40.2%)	81 (10.7%)	-
Age > 65 years	17.5%	23.7%	18.5%	0.127
ECOG > 2	11.3%	8.6%	9.1%	0.514
Hb < 90 g/L	31.0%	46.7%	55.6%	<0.001
PLT < 100 × 10^9^/L	10.1%	15.9%	16.0%	0.055
Plasma cell > 60% in the bone marrow	22.6%	28.9%	30.9%	0.100
Elevated LDH ^1^	11.5%	20.1%	24.1%	0.002
Renal failure ^2^	13.7%	15.8%	26.3%	0.022
ISS 3	40.7%	49.7%	60.5%	0.002
Hypodiploidy	3.0%	11.1%	14.7%	<0.001
Complex karyotype	6.1%	16.9%	19.1%	<0.001
13q abnormality by conventional karyotype	1.3%	7.7%	11.6%	<0.001
BTZ treatment	76.1%	75.0%	77.8%	0.861
First-line ASCT	28.5%	27.3%	21.0%	0.389
Early progression ^3^	18.5%	28.6%	45.7%	<0.001
Early death ^4^	12.4%	15.1%	33.3%	<0.001

^1^ LDH > 247 U/L. ^2^ Renal failure was defined as a serum creatinine concentration greater than 2 mg/dL or a creatinine clearance rate less than 40 mL/min/1.73 m^2^. ^3^ The percentage of patients who progressed 18 months after diagnosis. ^4.^ The percentage of patients who were dead 2 years after diagnosis.

**Table 4:** Multivariate analysis of ISS, HBDH HRCA, and patient-related features in MM.

	PFS		OS	
	HR	*p*	HR	*p*
No hit	-	-	-	-
One hit	1.60	<0.001	1.40	0.014
Double hit	2.24	<0.001	2.30	<0.001
ISS 3	1.13	0.219	1.69	<0.001
Elevated LDH	1.48	0.002	1.57	0.003
Age > 65 years	1.40	0.005	1.86	<0.001

## Data Availability

The datasets generated and/or analyzed during the current study are available from the corresponding author at angang@ihcams.ac.cn upon reasonable request.

## Supplementary Materials

The following supporting information can be downloaded at: https://www.mdpi.com/article/10.3390/cancers17162703/s1, Supplementary Figure S1: Flow chart showing the patient screening process followed in this study. Supplementary Figure S2: Kaplan–Meier analysis of multi-hit MM defined by the IMS/IMWG CGS system. Supplementary Figure S3: Kaplan–Meier analysis of multi-hit MM defined by different standards. Supplementary Figure S4: Kaplan–Meier analysis of the effect of del(1p) on each HRCA subgroup defined by the presence of t(4;14), t(14;16), t(14;20), gain(1q), and del(17p). Supplementary Figure S5: The survival impact of del(13q) on patients with or without high-risk cytogenetic abnormalities (HRCAs). Supplementary Figure S6: Kaplan–Meier analyses of patients with different types of IgH translocations and those with t(11;14) and/or secondary HRCAs. Supplementary Figure S7: The prognostic value of t(4;14) in the whole cohort, bortezomib (Btz) or non-bortezomib (non-Btz) treatment arm, high-risk cytogenetic abnormalities (HRCAs), or no HRCA subgroup. Supplemental Table S1. Baseline characteristics of 1122 newly diagnosed MM patients. Supplemental Table S2. Multivariate survival analysis of high-risk cytogenetic aberrations in 757 patients with complete CA data. Supplemental Table S3. The co-occurrency of cytogenetic aberrations with del(13q) in newly diagnosed multiple myeloma patients. Supplemental Table S4. The co-occurrency of secondary cytogenetic aberrations with t(11;14) in newly diagnosed multiple myeloma patients.
