# Supplementary material for: Development of a Cytogenetic Double-Hit Model for Survival Prediction in Multiple Myeloma
Source: Cancers (Basel). 2025 Aug 20;17(16):2703. doi: 10.3390/cancers17162703 (PMC12384134; doi:10.3390/cancers17162703)
Supplement: Supplementary file 1 [file cancers-17-02703-s001.zip › cancers-3813628-supplementary.pdf]

Supplementary data for

Development of a Cytogenetic Double-Hit Model for Survival Prediction in Multiple Myeloma

Chenxing Du [1,2,†], Jian Cui [1,2,3,†], Jingyu Xu [1,2,3], Wenqiang Yan [1,2], Lingna Li [1,2], Weiwei Sui [1,2], Shuhui Deng [1,2], Shuhua Yi [1,2], Yan Xu [1,2], Chengwen Li [1,2], Jiawei Zhao [1,2], Dehui Zou [1,2], Lugui Qiu [1,2] and Gang An [1,2,*]

Contents:

Supplementary table 1-4. Supplementary figure 1-7.

Supplemental Table S1. Baseline characteristics of 1122 newly diagnosed MM patients.

Characteristic	N = 1122
**Age, years, median (IQR)**	60 (50-66)
**Male, n (%)**	557 (56)
**Paraprotein type, n (%)**	
IgG	548 (48.8)
IgA	274 (24.4)
IgD	60 (5.3)
Light chain only	205 (18.3)
Missing	18 (1.6)
**Light chain type, n (%)**	
Lambda	570 (50.8)
Kappa	517 (45.2)
Missing	23 (2.0)
**ISS stage, n (%)**	
1	204/1113 (18.3)
2	386/1113 (34.7)
3	523/1113 (47.0)
**R-ISS stage, n (%)**	
1	143/1059 (13.5)
2	694/1059 (65.5)
3	231/1059 (28.1)
**Hb, g/dL, median (IQR)**	9.9 (8.0-12.3)
**Platelets, $\times 10^9$/L, median (IQR)**	178 (118-238)
**Serum creatinine, umol/L, median (IQR)**	85 (63-121)

**LDH, units/L, median (IQR)**	162 (136-218)
**B2M, ug/mL, median (IQR)**	4.3 (3.3-8.8)
**First-line therapy, n (%)**	
PI-based	685 (61.1)
IMiDs-based	263 (23.4)
PI+IMiDs-based	149 (13.3)
Others	26 (2.3)
**Upfront ASCT, n (%)**	249 (22.2)

Abbreviations: *IQR*, interquartile range; *ISS*, International Staging System; *R-ISS*, Revised International Staging System; *Hb*, hemoglobin; *LDH*, lactate dehydrogenase; *B2M*, β2-microglobulin; *HRCA* high-risk chromosome abnormality.

Supplemental Table S2. Multivariate survival analysis of high-risk cytogenetic aberrations in 757 patients with complete CA data.

Cytogenetic aberrations	PFS		OS	
	HR	*P*	HR	*P*
t(4;14)	0.98	0.92	0.81	0.29
t(14;16)	2.36	**0.002**	1.71	0.13
del(13q)	0.99	0.98	1.04	0.79
del(17p)	1.74	**<0.001**	1.80	**0.003**
gain(1q)	1.68	**<0.001**	1.60	**0.001**
del(1p)	0.88	0.56	1.14	0.61
Hypodiploidy[1]	1.05	0.79	1.41	0.22
CK[2]	1.71	**0.01**	1.37	0.18

1. Hyperdiploidy was defined as >46 chromosomes by metaphase cytogenetics, typically showing gains of multiple odd-numbered chromosomes.

2. CK, complex karyotype, defined as the presence of two or more cytogenetic abnormalities identified through conventional metaphase cytogenetics.

Supplemental Table S3. The co-occurrency of cytogenetic aberrations with del(13q) in newly diagnosed multiple myeloma patients.

	del(13q) negative N=430	del(13q) positive N=327	*P*
t(4;14)	6.7% (29)	31.2% (102)	<0.001
t(14;16)	2.3% (10)	5.5% (18)	0.031
t(11;14)	20.2% (87)	10.4% (34)	<0.001
del(17p)	5.3% (23)	15.9% (52)	<0.001
gain(1q)	33.3% (143)	58.7% (192)	<0.001
del(1p)	4.2% (18)	10.1% (33)	0.002
Hypodiploidy	4.6% (16/346)	11.5% (30/261)	0.002
Complex Karyotype	10.4% (36/346)	13.8% (36/261)	0.207

Supplemental Table S4. The co-occurrency of secondary cytogenetic aberrations with t(11;14) in newly diagnosed multiple myeloma patients.

	t(11;14) negative N=636	t(11;14) positive N=121	*P*
del(13q)	46.1% (293)	28.1% (34)	<0.001
del(17p)	10.8% (69)	5.0% (6)	0.047
gain(1q)	46.1% (293)	34.7% (42)	0.022
del(1p)	7.4% (47)	3.3% (4)	0.115
Any HR sCA	51.7% (329)	40.5% (49)	0.015
Hypodiploidy	7.2% (37/511)	9.4% (9/96)	0.527
Complex Karyotype	11.7% (60/511)	12.5% (12/96)	0.863

Supplemental Figure S1. Flow chart showing the patients screening process of this study.

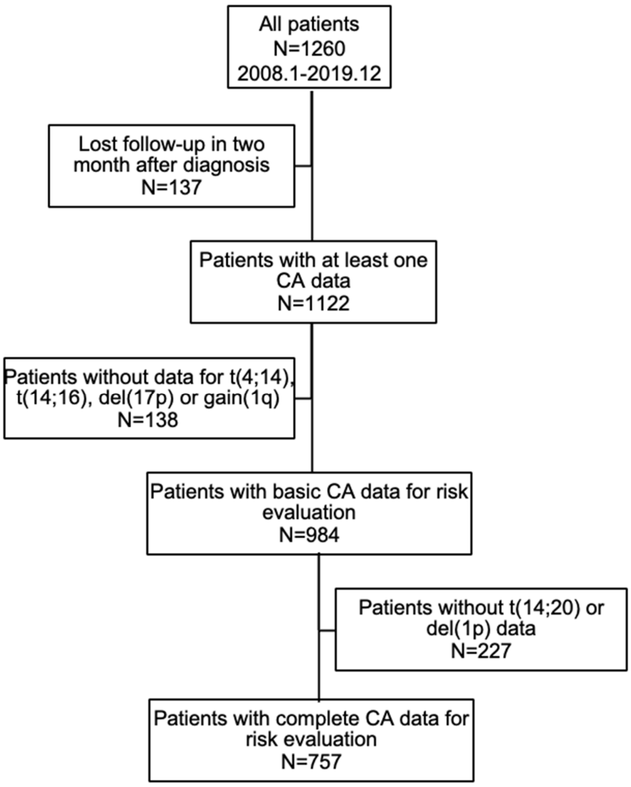

Supplementary Figure S2. Kaplan-Meier analysis of multi-hit MM defined by the IMS/IMWG CGS system.

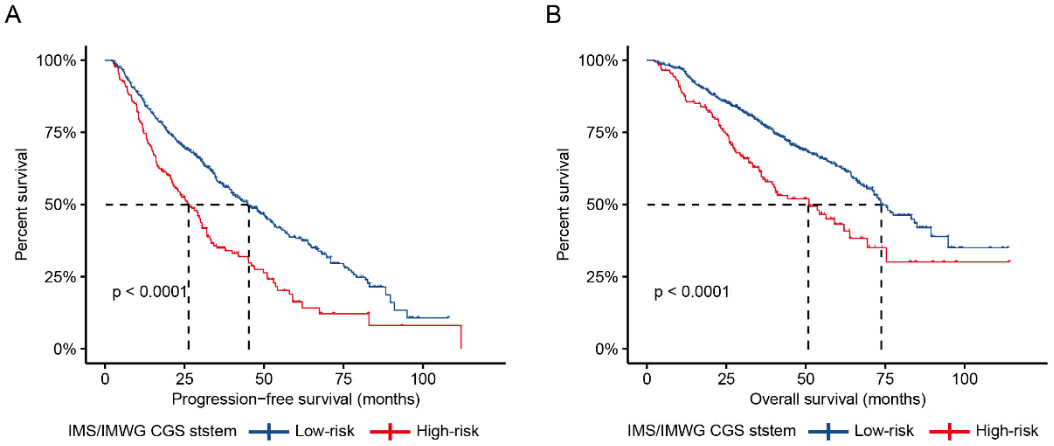

A, B. The Kaplan-Meier curves of PFS or OS for patients with different multi-hit statuses defined by the IMS/IMWG CGS system.

Supplementary Figure S3. Kaplan-Meier analysis of multi-hit MM defined by different standards.

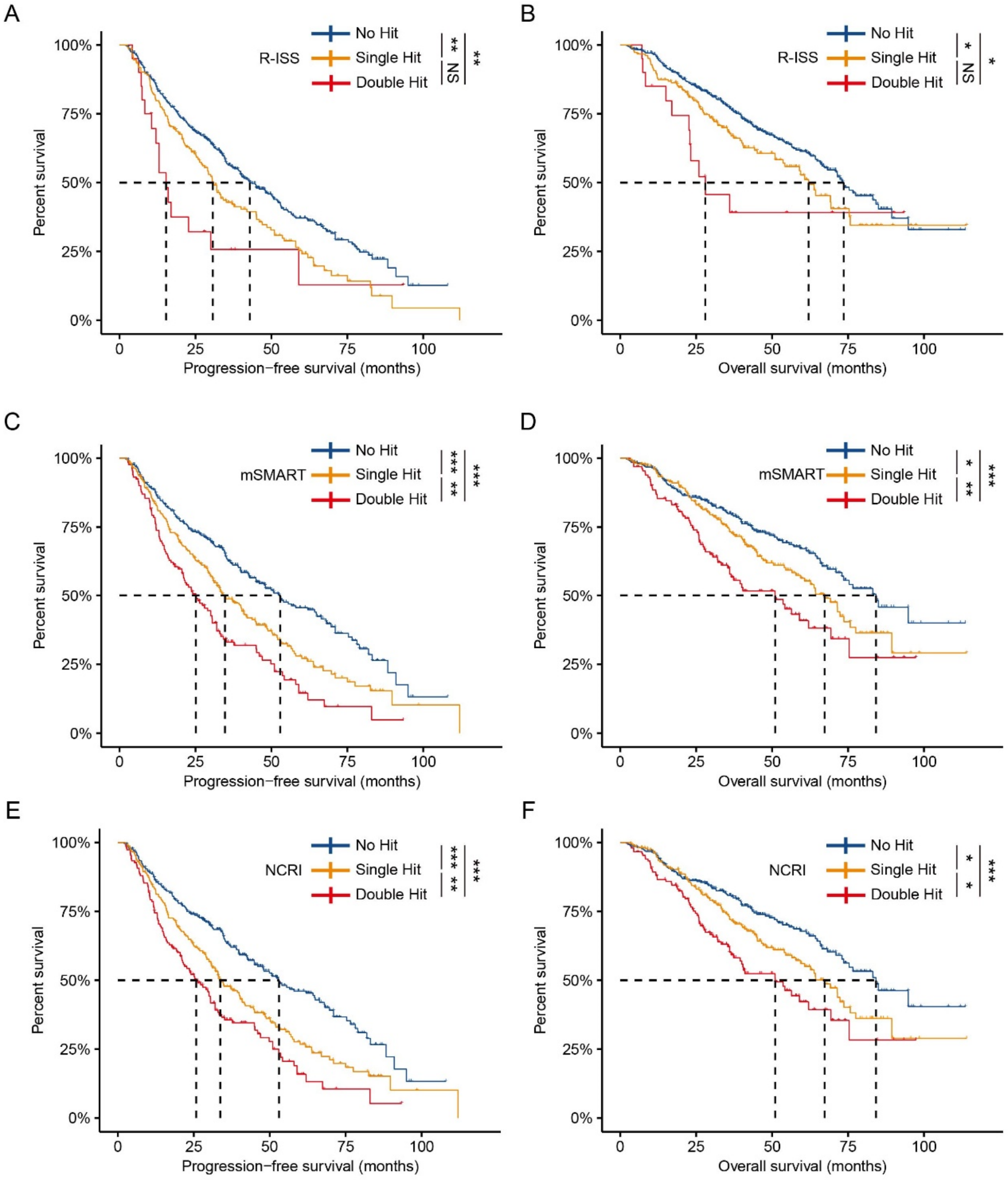

A, B. The Kaplan-Meier curves of PFS or OS for patients with different multi-hit statuses defined by R-ISS. **C, D.** The Kaplan-Meier curves of PFS or OS for patients with different multi-hit statuses defined by mSMART. **E, F.** The Kaplan-Meier curves of PFS or OS for patients with different multi-hit statuses defined by NCRI.

Supplementary Figure S4. Kaplan-Meier analysis of the effect of del(1p) on each HRCA number subgroup defined by the presence of t(4;14), t(14;16), t(14;20), gain(1q) and del(17p).

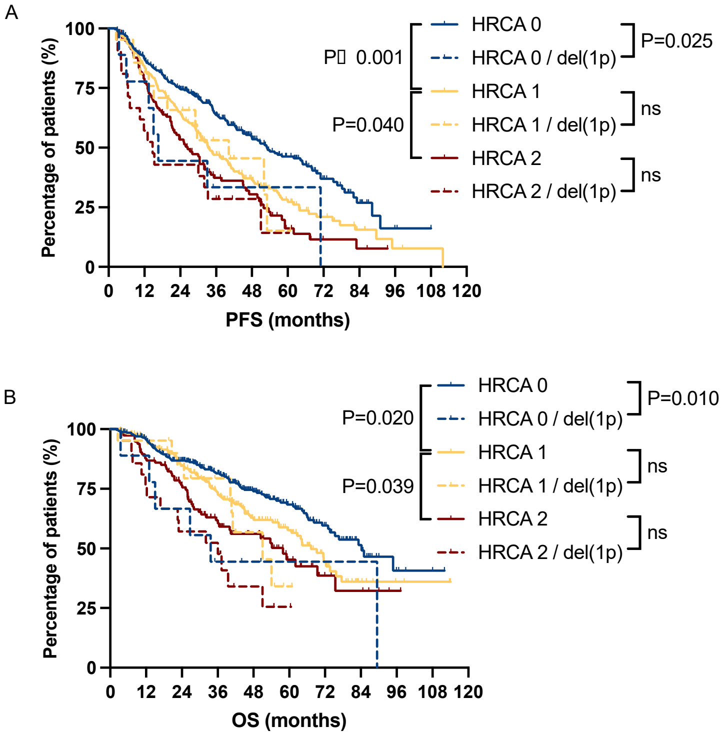

A, B. The Kaplan-Meier curves of PFS or OS of the effect of del(1p) on each HRCA number subgroup defined by the presence of t(4;14), t(14;16), t(14;20), gain(1q) and del(17p).

Supplementary Figure S5. The survival impact of del(13q) on patients with or without high-risk cytogenetic abnormalities (HRCA).

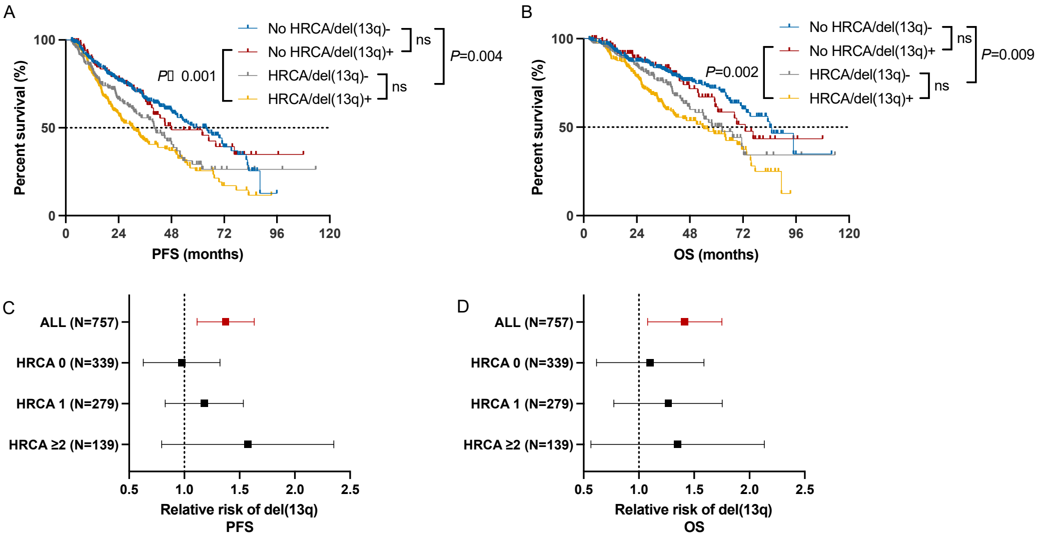

A, B. The Kaplan-Meier curves of PFS or OS of patients with del(13q) and/or HRCA.

C, D. Subgroup relative risk analysis of del(13q) stratified by the number of HRCA.

Supplementary Figure S6. Kaplan-Meier analyses of patients with different types of IgH translocations and those with t(11;14) and/or secondary HRCA.

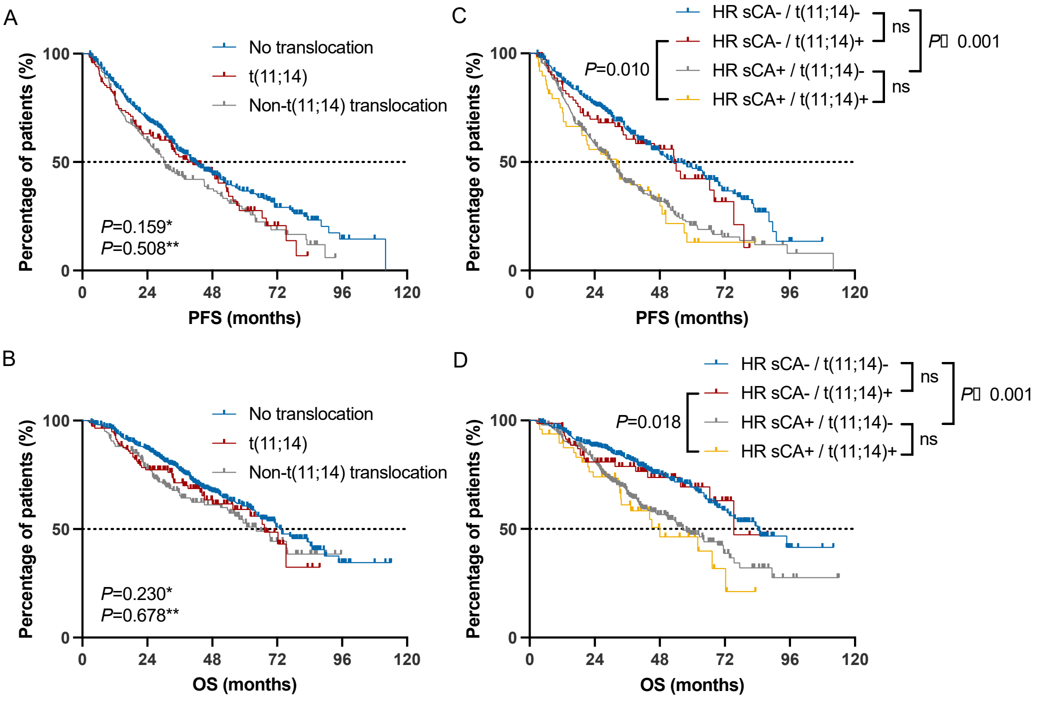

A, B. PFS or OS curves of patients with no IgH translocation, t(11;14) or non-t(11;14) IgH translocations. **C, D.** The PFS or OS curves of patients with t(11;14) and/or secondary HRCA.

Supplementary Figure S7. The prognostic value of t(4;14) in the whole cohort (A, B), bortezomib (Btz) or non-bortezomib (non-Btz) treatment arm (C, D), high-risk cytogenetic abnormalities (HRCA) or no HRCA subgroup (E, F).

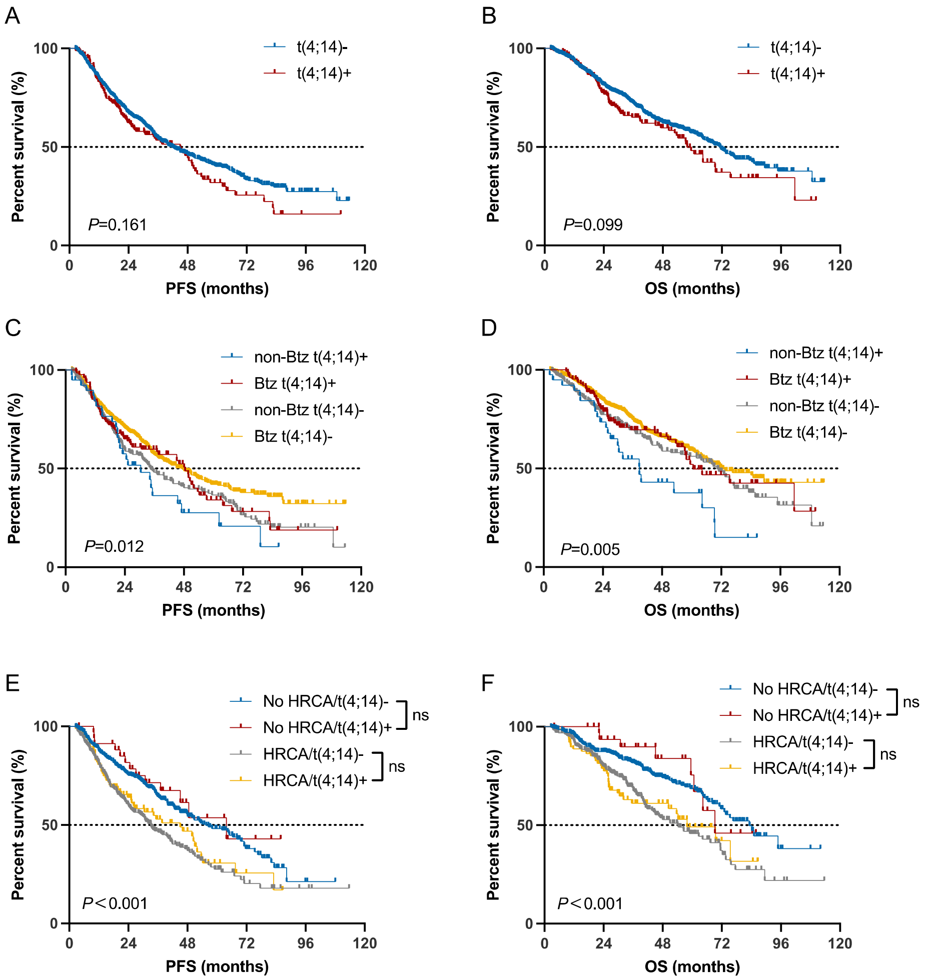

HRCA: any one of t(14;16), gain(1q), del(1p) and del(17p)
